# Evaluation of Turkish Dentists’ Approach to Over-the-Counter Whitening Agents: A Questionnaire-Based Study

**DOI:** 10.7759/cureus.60602

**Published:** 2024-05-19

**Authors:** Mine Başan Tosun, Batu Can Yaman, Ozge Celiksoz, Hatice Tepe

**Affiliations:** 1 Department of Restorative Dentistry, Eskişehir Osmangazi University Faculty of Dentistry, Eskişehir, TUR

**Keywords:** whitening agents, restorative dentistry, survey analysis, over-the-counter, tooth whitening

## Abstract

Aim: Over-the-counter teeth-whitening products have become popular in accordance with the increasing demands of patients. These products can also be recommended to patients by dentists. The aim was to determine the rates of recommendation of over-the-counter teeth whitening products by dentists in Turkey divided into different categories for their patients and to interpret them based on a cause-and-effect relationship.

Material and methods: After obtaining ethics committee approval, the survey questions were created using Google Forms (Google Inc., Mountainview, CA) and delivered to dentists via social media. Dentists who signed the informed consent form completed the survey. Within the study’s scope, statistical analyses were conducted using IBM SPSS Statistics for Windows, version 26.0 (IBM Corp., Armonk, NY). Values were expressed as frequency (n) and percentage (%). A chi-square test was used to compare participants’ teeth-whitening recommendations according to their demographic characteristics. The statistical significance level was accepted as p <0.05 throughout the study.

Results: A total of 57.9% of dentists working in Turkey did not recommend over-the-counter teeth-whitening products to their patients. When the answers to the question “Which whitening products sold on the market do you recommend to your patients?” were compared according to the participants' place of work, there was a statistically significant difference between the place of work and the recommended whitening product (p <0.05). Participants working in the private sector answered “I do not recommend” at a higher rate than participants working in the public sector. Among those who recommended toothpaste, more dentists with five or fewer years of experience recommended toothpaste to their patients than dentists with greater than five years of experience. Additionally, more dentists working in the public sector recommended toothpaste to their patients than dentists working in the private sector, and more specialist dentists recommended toothpaste to their patients than general practitioner dentists.

Conclusion: Most dentists in Turkey do not recommend over-the-counter teeth-whitening products to their patients. Among those who recommend such products, toothpaste has been determined to be the first choice. The results of this study may raise awareness among dentists about over-the-counter teeth-whitening products and encourage new studies.

## Introduction

People with high self-confidence can establish healthier human relationships and be happier in their social lives. Appearance is also very significant in having high self-confidence. This is why people consult dentists to make their teeth look whiter and their smiles more aesthetic. Vital teeth whitening has been performed in clinics or at home under the supervision of a dentist for years. Additionally, because of technological developments and the increasing presence of social media in our lives, teeth-whitening options that patients can obtain and use themselves are becoming popular today [1,2].

Over-the-counter (OTC) products, also known as OTC whitening products, are low-cost whitening products that patients can obtain from pharmacies, markets, the internet, and cosmetic stores and apply themselves without a physician prescribing them [3]. Generally, hydrogen peroxide rates vary between 5.3% and 6.5%, and the total duration of use is between 10 days and two weeks with a twice-a-day application. Toothpaste, whitening mouthwashes, paint-on system products applied to the teeth with a brush, whitening strips, and pens and trays containing light-activated whitening gel are included in this group [1,4].

The most successful whitening results are achieved when OTC products are used to maintain the color of previously professionally whitened teeth. In particular, dentists recommend toothpaste that does not contain abrasives and that prevents recoloring during and after whitening [5].

Most of the market for OTC products consists of toothpaste. This outcome is due to the proliferation of places where such products are sold, the success of marketing companies, and the ease of access to such products. While whitening toothpastes generally do not contain peroxide agents, they have whitening effects thanks to their abrasive ingredients such as alumina, silica, dicalcium phosphate dihydrate, and enzymes. Their effectiveness is limited to stripping extrinsic stains; they do not cause tooth discoloration [1]. Even those OTC products containing very low amounts of hydrogen peroxide have minimal whitening effectiveness because the exposure time to the toothpaste and whitening agent is very short [6,7].

Whitening mouthwashes are newly introduced products containing 1.5% hydrogen peroxide and sodium hexametaphosphate to prevent new stains [1]. Their effects can last for three months, and they can lighten the tooth tone by one to two shades. Because their irritation power is high, long-term use is contraindicated [1,8,9].

Whitening strips are a popular OTC product. Disposable plastic strips contain hydrogen peroxide gel at a concentration of 5% and 14% and are placed on the teeth. The peroxide rate they contain is higher than the average of other OTC products. They should be applied twice a day for 30 minutes for two weeks [6,7].

Paint-on systems are those in which whitening occurs by applying peroxide-containing gel to the teeth with a brush and waiting. Despite these systems’ peroxide content, insufficient whitening occurs because of the short contact time of the agent with the teeth. New products that are in pencil form and applied at night are also included among the paint-on systems [10,11].

Trays with whitening agents placed in them function by activating a whitening gel containing 9% hydrogen peroxide inside the trays, which the patient applies themselves to with a light-emitting diode (LED) light. However, since the application is not performed under the supervision of a physician or with a professional diagnosis, extreme caution should be exercised [12]. In the literature, problems such as acute pain, soft oral tissue damage, and malocclusion have been reported due to non-customized and non-adaptable trays in which whitening gels are placed and applied. [9,12]. Malocclusion is one of the main causes of temporomandibular joint (TMJ) problems [13]. For this reason, patients may encounter TMJ problems if they use aligners that are not compatible with their own dentition without dental control [14].

Various clinical and in vitro studies on tooth whitening have been conducted from the past to the present. The number of studies on OTC products is insufficient. New studies are needed regarding these products, which patients can obtain without medical supervision and whose use has become widespread today. As professionals, we need to obtain more information about OTC whitening products through new studies for the public's health. Today, dentistry surveys play a guiding role in determining patient-physician relationships and material preferences. They are useful methods for measuring existing information. This study was aimed at investigating the relationship between dentists' professional experience, status, the sectors they work in, and their preferences for OTC products through a questionnaire survey.

## Materials and methods

Aim of the study

This study aimed to comparatively evaluate the rates of active dentist recommendations of OTC teeth whitening products to patients in Turkey. The approaches and preferences of specialist and general dentists working in the public and private sectors, with different professional experiences, were investigated.

Research methodology

Survey questions were created using Google Forms (Google Inc., Mountainview, CA). After the approval of the Eskişehir Osmangazi University Non-interventional Clinical Research Ethics Committee, Eskişehir (approval number: E-25403353-050.99-2400075290), it was delivered to dentists in Turkey. The Turkish Dental Association’s database was used for this purpose. Various social media tools and email were used to send the survey. The responses received from the participants were tabulated using Microsoft Excel (Microsoft Corp., Redmond, WA).

This was a web-based, cross-sectional study conducted on a voluntary basis. Each dentist completed the survey after signing a letter informing them about their rights and filling out the informed consent form. Participants were informed that no personal data would be collected and that the data they provided would be anonymized. According to the results of power analysis performed with the G Power program (G*Power 3.1 software; Heinrich Heine University, Düsseldorf, Germany), the chi-squared (x2) test and the goodness-of-fit test. Contingency table analysis was performed at 20 degrees of freedom, a margin of error (α) = 0.05, effect size (w) = 0.35, power value (1-β) = 0.95, and it was determined that a minimum of 251 samples were sufficient in total. The study was terminated after 303 people had answered the survey.

Participant selection

Dentists who had completed their undergraduate education and were actively working in the public or private sector in all provinces of Turkey, who were specialists or practitioners, and who had professional experience or no experience were randomly selected for the study. The questionnaire was delivered via e-mail to dentists registered with the Turkish Chamber of Dentists. The study was completed by dentists who answered the questionnaire. Dentists who were continuing their undergraduate education or were interns were excluded.

Questionnaire

This web-based questionnaire, developed by the researchers, was designed after a comprehensive literature review of previous studies. To verify the clarity and reliability of the questionnaire, a group of 25 people representing the study population participated in a pilot test. It consisted of eight questions in total. The first seven questions pertained to demographic information about dentists’ age, gender, title, specialization status, length of professional experience, graduation year, and place of work. The remaining question was multiple choice: “Which whitening products sold on the market do you recommend to your patients?” The options were “I do not recommend,” “Whitening toothpaste,” “Whitening pens and strips,” “Plate containing light-activated whitening gel,” and “Whitening mouthwash.” This question had 335 answers and was analyzed in this form. All data and results were interpreted in association with the necessary statistical analyses.

Data analysis

Within the study’s scope, statistical analyses were conducted using IBM SPSS Statistics software for Windows version 26.0 (IBM Corp., Armonk, NY). Values were expressed as frequency (n) and percentage (%). Pearson’s chi-squared test was performed to compare participants’ OTC teeth-whitening product recommendations according to their demographic characteristics (specialization status, years of professional experience, employed institution). The statistical significance level was accepted as p <0.05 for the entire study.

## Results

A total of 303 participants’ answers were examined. General demographic information about the participants is shown in Table 1. A total of 37.6% of the participants were male dentists, and 62.4% were female dentists. Regarding their age, 44.9% were in the 25-30 age group, 19.5% were in the 20-25 age group, 21.8% were in the 30-35 age group, 5% were in the 35-40 age group, and 8.9% were in the over-40 age group. Further, 59.1% of the participants were dentists, 25.4% were doctoral/specialist dentists, and 15.5% were specialist/doctoral students. Regarding their specialization status, 58.1% were general dental practitioners, and 41.9% were specialist dentists. A total of 5.3% of the participants had graduated before the year 2000, 8.9% had graduated between 2000 and 2010, 60.1% had graduated between 2010 and 2020, and 25.7% had graduated after 2020. A total of 54.8% of the participants had been working as dentists for five years or fewer, whereas 45.2% had more than five years of professional experience. A total of 31.4% of the participants worked in the public sector, whereas 68.6% worked in the private sector.

**Table 1:** Basic descriptive information about the participants n(%): the categorical variables are expressed in frequencies and percentages.

		n	%
Gender	Male	114	37.6
	Female	189	62.4
Age (years)	20–25	59	19.5
	Between 25 and 30	136	44.9
	Between 30 and 35	66	21.8
	Between 35 and 40	15	5.0
	40+	27	8.9
Title	General dentist	179	59.1
	Doctorate/Specialized dentist	77	25.4
	Specialization/PhD student	47	15.5
Specialization status	Specialist dentist	127	41.9
	General dentist	176	58.1
Graduation year	Before 2000	16	5.3
	2000–2010	27	8.9
	2010–2020	182	60.1
	Post 2020	78	25.7
Years of professional experience	Five years or fewer	166	54.8
	Over five years	135	45.2
Employed institution	Public sector	95	31.4
	Private sector	208	68.6

Participants were asked about the whitening product they recommended to their patients. For the multiple-choice question, 335 answers were obtained from 303 participants. A total of 57.9% of participants stated that they did not recommend a teeth-whitening product; 42.4% stated that they recommended whitening toothpaste; 4% recommended whitening pens and strips; 3.3% recommended a plaque containing a light-activated whitening gel; and 3.3% recommended whitening mouthwash (Table 2).

**Table 2:** Recommended OTC whitening products n(%): the categorical variables are expressed in frequencies and percentages OTC: over-the-counter

		Responses		Respondent %
		n	%	
Recommended OTC whitening products	I do not recommend	175	52.20%	57.90%
	Whitening toothpaste	128	38.20%	42.40%
	Whitening pens and strips	12	3.60%	4.00%
	Plaque with light-activated Whitening gel	10	3.00%	3.30%
	Whitening mouthwash	10	3.00%	3.30%
	Total	335	100.00%	110.90%

Participants were asked, “Which whitening products sold on the market do you recommend to your patients?” When the answers to the question were compared in terms of the expertise of the participants, no statistically significant difference was found between expertise and recommended whitening products (p >0.05). However, the most fundamental difference was in the recommendation of whitening toothpaste. More specialist dentists recommended whitening toothpaste than general dentists (Table 3).

**Table 3:** Analyzing the answers to the question “Which whitening products sold on the market do you recommend to your patients?” according to specialization status n(%): the categorical variables are expressed in frequencies and percentages; *Chi-square test is used, and p <0.05 is considered statistically significant. OTC: over-the-counter

		Specialization status		p
		General dentist n (%)	Specialist dentist n (%)	
Recommended OTC whitening products	I do not recommend	104 (59.1%)	71 (56.3%)	0.128
	Whitening toothpaste	70 (39.8%)	58 (46.0%)	
	Whitening pens and strips	6 (3.4%)	6 (4.8%)	
	Plaque with light-activated whitening gel	7 (4.0%)	3 (2.4%)	
	Whitening mouthwash	2 (1.1%)	8 (6.3%)	

Participants were asked, “Which whitening products sold on the market do you recommend to your patients?” When the answers to the question were compared according to participants’ professional experience in dentistry, the results showed that there was no statistically significant difference between professional experience in dentistry and recommended whitening products (p >0.05). However, participants who had been working in the field of dentistry for five years or less recommended whitening toothpaste at a higher rate. More participants who had been working in the field for five years and above responded that they did not recommend OTC products than participants who had been working in the field for five years or less (Table 4).

**Table 4:** Examination of the answers to the question “Which whitening products sold on the market do you recommend to your patients?” according to years of professional experience in dentistry n(%): the categorical variables are expressed in frequencies and percentages. OTC: over-the-counter

		Years of professional experience		p
		Five years or fewer n (%)	Over five years n (%)	
Recommended OTC whitening products	I do not recommend	91 (55.2%)	84 (61.3%)	0.576
	Whitening toothpaste	74 (44.8%)	54 (39.4%)	
	Whitening pens and strips	7 (4.2%)	5 (3.6%)	
	Plaque with light-activated whitening gel	7 (4.2%)	3 (2.2%)	
	Whitening mouthwash	4 (2.4%)	6 (4.4%)	

Participants were asked, “Which whitening products sold on the market do you recommend to your patients?” When the answers to the question were compared according to participants’ workplaces, the results showed a statistically significant difference between workplaces and recommended whitening products (p <0.05). Participants working in the private sector answered “I do not recommend” at a higher rate than participants working in the public sector. At the same time, participants working in the public sector were more likely to recommend whitening toothpaste, whitening pens and strips, and whitening mouthwash than participants working in the private sector (Table 5).

**Table 5:** Examination of answers to the question “Which whitening products sold on the market do you recommend to your patients?” according to participants’ place of work n(%): the categorical variables are expressed in frequencies and percentages OTC: over-the-counter

		Employed institution		p
		Public sector n (%)	Private sector n (%)	
Recommended OTC whitening products	I do not recommend	50 (53.2%) ^a^	125 (60.1%) ^b^	0.001*
	Whitening toothpaste	46 (48.9%) ^a^	82 (39.4%) ^b^	
	Whitening pens and strips	7 (7.4%) ^a^	5 (2.4%) ^b^	
	Plaque with light-activated whitening gel	4 (4.3%)	6 (2.9%)	
	Whitening mouthwash	9 (9.6%) ^a^	1 (0.5%) ^b^	

## Discussion

People want whiter teeth for reasons such as developments in digital photography and the spread of social media and advertising. Thus, people resort to different methods to whiten their teeth. The demand is increasing for whitening products that can be sold without a prescription and can be easily obtained offline and online.

For patients, going to the dentist for whitening treatment may seem expensive and time-consuming. Instead, patients can obtain and apply whitening products they see advertised on social media without the need for a dentist [2]. In a survey conducted in Saudi Arabia, 30% of people reported that they learned about whitening from dentists, and 47.5% reported that they learned about it from advertisements and social media. In the results of a survey conducted in Malaysia, 30.6% of patients reported that they used OTC teeth whitening products without consulting a physician [15].

The high demand from patients for OTC teeth whitening products has led to an increase in the number of studies performed on this subject. Although surveys have been conducted among patients, surveys evaluating dentists’ knowledge and preferences are insufficient. In the current study, 42.1% of dentists were found to recommend OTC teeth whitening products to patients. According to the results of a survey investigating the frequency of the use of OTC teeth whitening products, 86% of patients used them voluntarily, and 14% used them based on their dentist’s advice [15]. In a survey conducted in Brazil, where vital teeth whitening indications were examined, only 5.2% of dentists stated that they preferred OTC teeth whitening products as their first choice [16].

In the current study, dentists’ OTC teeth-whitening product preferences were divided into different categories and compared. Specialist dentists advised OTC teeth whitening products to their patients more than general practitioners. The same was true for dentists with more than five years of experience than those with five or fewer years of experience and for dentists working in the public sector rather than in the private sector. According to the survey results in Brazil, specialist dentists recommended OTC whitening products more than general dentists. Similar to the current study, the preference for OTC whitening products increased as dentists’ professional experience decreased [16].

Studies have advocated that OTC whitening products should include home whitening methods. The effectiveness of such whitening products increases over time, and they can be used with a physician’s advice [9,17]. However, there is no consensus on this issue. According to a few studies, the whitening effects of OTC products are not adequate, and such products are not safe when applied without a dentist’s supervision [18,19]. A comparison of home whitening products and OTC products with approximately the same concentration showed that OTCs can lead to problems such as sensitivity, gum injuries, and malocclusion [20].

Toothpastes cover more than 50% of the OTC teeth-whitening product market. Toothpastes are the most easily available OTC whitening products because they are believed to eliminate the inequity of public access to whitening materials. In the present study, dentists who recommended OTC teeth whitening products to their patients largely recommended “whitening toothpaste." According to the results of many surveys, the most preferred product by the public is whitening toothpaste [17,21].

Many studies have shown that toothpastes are not as effective as whitening products, as claimed. Most toothpastes do not contain hydrogen peroxide or carbamide peroxide. Additionally, toothpastes are popularly believed to whiten teeth because of their various light illusions and abrasive effects. However, in a study investigating whitening toothpaste’s effect on tooth color lightening, it was reported that because of its abrasive properties, the toothpaste removed external discolorations without causing any real change in tooth color [7]. In a review investigating toothpastes’ whitening effects, toothpastes were found to be more effective than placebo and non-whitening pastes. However, their main area of effectiveness was perceived to be the removal of external colorings [3].

Whitening strips, which have become very common as OTC whitening products recently, appear frequently on social media and in advertisements. They are the most studied OTC whitening products after toothpastes regarding their whitening effectiveness [3]. According to the current study’s results, the rate of dentists in Turkey who recommended whitening strips to their patients was 4%. In another study, 5% of dentists recommended whitening strips [16].

In a clinical study, whitening patches containing 6.5% hydrogen peroxide were applied to patients with tetracycline discoloration for two months. A 66% improvement was observed. No side effects other than short-term and temporary sensitivity were observed. It was concluded that the results would have been more positive if the application had been made for six months [22]. In clinical studies comparing traditional 10% carbamide peroxide plaque treatment with whitening patches containing 5.3% and 6.5% hydrogen peroxide, the color change of the whitening patches was found to be similar or higher [9,20]. Researchers associated this result with the fact that the concentration of hydrogen peroxide separated from 10% carbamide peroxide is less than the concentration of hydrogen peroxide found in whitening strips. Another study compiled the literature on four different OTC whitening products. All the literature shows that whitening strips are more effective than other OTC whitening agents. The reason strips provide more effective results than other OTC products is presumably that the strips act as a retention and barrier on the tooth [3]. A systematic review showed that carbamide peroxide plaque treatment had better effectiveness compared to whitening strips [23].

A literature review of OTC whitening products in 2020 showed a lack of evidence about the effectiveness of OTC products applied to plates containing light-activated gel [3]. In this study, the rate of dentists recommending light-activated gel-containing aligners to their patients was found to be 3%. The fact that the percentage of dentists is lower than others in recommending this product may be due to a lack of knowledge. In the future, in vivo and in vitro studies on these products should be increased and included in survey studies.

There are many studies on whitening mouthwashes, and the results are generally in favor of their whitening effectiveness. A study comparing two whitening mouthwashes in vitro found that both mouthwashes effectively whitened teeth. However, the study emphasized that caution should be exercised, especially because these mouthwashes contained low pH [24]. According to the results of the study, which aimed to compare whitening mouthwash and whitening treatment with a 10% carbamide peroxide-containing tray, both provided effective whitening. However, home whitening was more successful [25].

In recent developments worldwide, the European Medicines Agency banned OTC tooth whitening products containing more than 6% hydrogen peroxide to prevent harm due to their unconscious use by the public [9,26] Additionally, the EU Council decided that whitening products containing 0.1%-6% hydrogen peroxide should only be sold to dentists [26]. Markets in European countries were severely affected by this decision and started to look for alternatives. Because the desired result could not be obtained with less than 0.1% hydrogen peroxide, other agents were developed to be added to OTC products. Chemicals such as sodium chloride and sodium carbonate were added to the products [4]. In an in vitro study conducted to find an alternative to hydrogen peroxide, an experimental tooth-whitening solution containing 0.1% hydrogen peroxide mixed with doping agents was used. The results were positive but needed further confirmation [19,27].

Teeth whitening treatment is known to cause tooth sensitivity in patients worldwide [28]. According to the results of a comprehensive survey conducted in Nigeria, 80.3% of patients who consulted dentists experienced tooth sensitivity as a result of teeth whitening [29]. This problem can also be encountered as a result of the use of OTC products. Among such products, the highest sensitivity is seen for toothpastes. This effect is enhanced by abrasive agents and by everyday use [1,6,7].

Scientists’ biggest concern today regarding OTC teeth whitening treatment is its unreliability and risk of harm to human life because of misuse. Additional studies are needed on this subject, and the results should be publicized. The public uses OTC whitening products without being informed about their harmful effects. According to the results of a large-scale survey conducted in Riyadh, more than half of the population did not know whether the OTC whitening products they used were safe or not [21]. According to the results of a study investigating the presence of heavy metals in whitening products in India, lead levels in three out of nine samples were found to be much higher than the permitted value. Moreover, the lead content was not mentioned on commercial products’ packaging [30]. New products are being introduced into the market every day. Their supervision is a problem because of social media’s involvement. Further, the number of studies on these products is insufficient. In order to reach meaningful conclusions about OTC teeth-whitening products over time, the current survey study should be expanded with more participants and more questions. The results obtained should be supported by in vivo and in vitro research.

Limitations

Interns who had not completed their undergraduate education were not included in this study. The study, which consisted of a total of eight questions, was terminated when the number of dentists reached 303. This study is a questionnaire-based survey. For more comprehensive results, the limitations of the study are that it was not supported by in vivo and in vitro studies, the number of questions, and the small number of participants.

## Conclusions

Surveys on tooth coloring and whitening have an important place in dentistry. The majority of dentists in Turkey do not recommend OTC whitening products to their patients. Among those who do, the most commonly recommended OTC whitening product is toothpaste. Specialized dentists in Turkey are more likely to recommend toothpaste to their patients than general practitioners. Additionally, dentists with five or fewer years of experience are more likely to recommend toothpaste to their patients than dentists with more than five years of experience, and dentists working in the public sector are more likely to recommend toothpaste to their patients than dentists working in the private sector.
